# A pilot study for intraocular pressure measurements based on vibroacoustic parameters

**DOI:** 10.1038/s41598-020-80321-1

**Published:** 2021-01-13

**Authors:** Deukha Kim, Youngbeen Chung, Yeji Yeon, Hyunsoo Cho, Han Woong Lim, Junhong Park, Won June Lee

**Affiliations:** 1 Department of Mechanical Engineering, Hanyang University College of Engineering, Seoul, Korea; 2 Department of Ophthalmology, Hanyang University College of Medicine, Wangsimni-ro 222, Seongdong-gu, Seoul, 04763 Korea; 3 Department of Ophthalmology, Hanyang University Seoul Hospital, Wangsimni-ro 222, Seongdong-gu, Seoul, 04763 Korea

**Keywords:** Glaucoma, Mechanical engineering

## Abstract

The present study aimed to identify vibroacoustic properties associated with intraocular pressure (IOP) changes and to suggest a new way to measure the IOP based on these properties. Ten ex vivo porcine eyeballs were used in this study. Each eyeball was fixated in a central hole of a Styrofoam block, and vibration applied to the Styrofoam block was transmitted to the eyeball. An accelerometer directly attached to the eyeball measured the vibration response. Excitations and measurements were performed for 1 s, and the excitation magnitude was varied for the same signal in repeat measurements. A 30-gauge needle was inserted into the anterior chamber of the eyeball to inject a balanced salt solution, and the height of the bottle was adjusted to adjust the IOP. A tonometer was used under identical conditions to measure the IOP five times, and the mean value was determined for further analyses. The measurements showed that the parameters resonance frequency and change in the magnitude of the vibration response (CMVR) increased with rising IOP values. The CMVR was highly correlated with the IOP (p-value < 0.0001). A linear mixed effects model (LMM) was used as a statistical analysis method. We confirmed that vibroacoustic properties of the eyeball are correlated with IOP changes. It is expected that the CMVR will serve as a new parameter for IOP measurements. Thus, in the future, continuous IOP measurements would be easily performed using the CMVR.

## Introduction

Intraocular pressure (IOP) measurement is a routine procedure conducted by eye care specialists. Increased IOP is the most important risk factor for the occurrence and progression of glaucoma^1,2^. Although glaucoma can develop regardless of IOP^2,3^, current treatments are primarily focused on lowering IOP. Therefore, IOP measurement is essential to determine the effectiveness of treatment and to monitor patients. Primary open-angle glaucoma and normal-tension glaucoma are both progressive optic neuropathies that differ by definition only in the untreated IOP which is > 21 mmHg in primary open-angle glaucoma and ≤ 21 mmHg in normal-tension glaucoma. Furthermore, IOP measurement is in glaucoma the most crucial tool to monitor treatment outcomes and disease progression.

An ideal tonometer yields accurate, reproducible, and repeatable measurements without influencing the IOP or injuring the eye. At present, different models of noncontact tonometer, Goldmann applanation tonometer (GAT), rebound tonometer, and Tono-Pen are being used in clinical practice^4–10^. Among various tonometers, the GAT is the most commonly used type of tonometer and regarded as the reference standard. However, since the plastic tonometer tip presses during slit-lamp biomicroscopy against the locally anesthetized cornea, it is difficult to use GAT in patients who are not compliant, such as children or bedridden patients. Moreover, the thickness of the central cornea may greatly influence GAT measurements^11,12^.

Since the IOP is not constant and fluctuates in a circadian rhythm, IOP measurements during normal activity or at night may provide useful information for glaucoma monitoring and treatment^13^. To assess the nychthemeral IOP, patients are admitted and IOP measurements occur every 2–3 h over a 24-h period. Although implantable or portable devices including contact lens tonometers^14,15^ and self-measurement rebound tonometers^4,9^ are commercially available, there is still an increasing need for new tonometers that are easier to use, more user-friendly, and can be employed for daily monitoring of patients outside clinical settings.

Vibroacoustic properties of an object can be used to determine its pressure. Previous studies have attempted to use acoustic wave vibration, a relatively noninvasive stimulation, to infer the biomechanical properties of ocular structures^16–18^. Previous studies report that corneal acoustic impedance^19^ as well as the wave speed in the cornea^20,21^ are correlated with the IOP. Other publications attempted to derive correlations between IOP and ocular resonance frequency^22,23^ or vibration reduction properties through experimental and theoretical approaches^24,25^.

To this end, the present study aimed to analyze various eyeball parameters in response to vibratory stimulation using an ex vivo porcine eye model to analyze the correlation of these parameters with IOP and to identify new parameters that may be used to determine the IOP.

## Results

### Variations of vibration responses with IOPs

**Figure 1:**
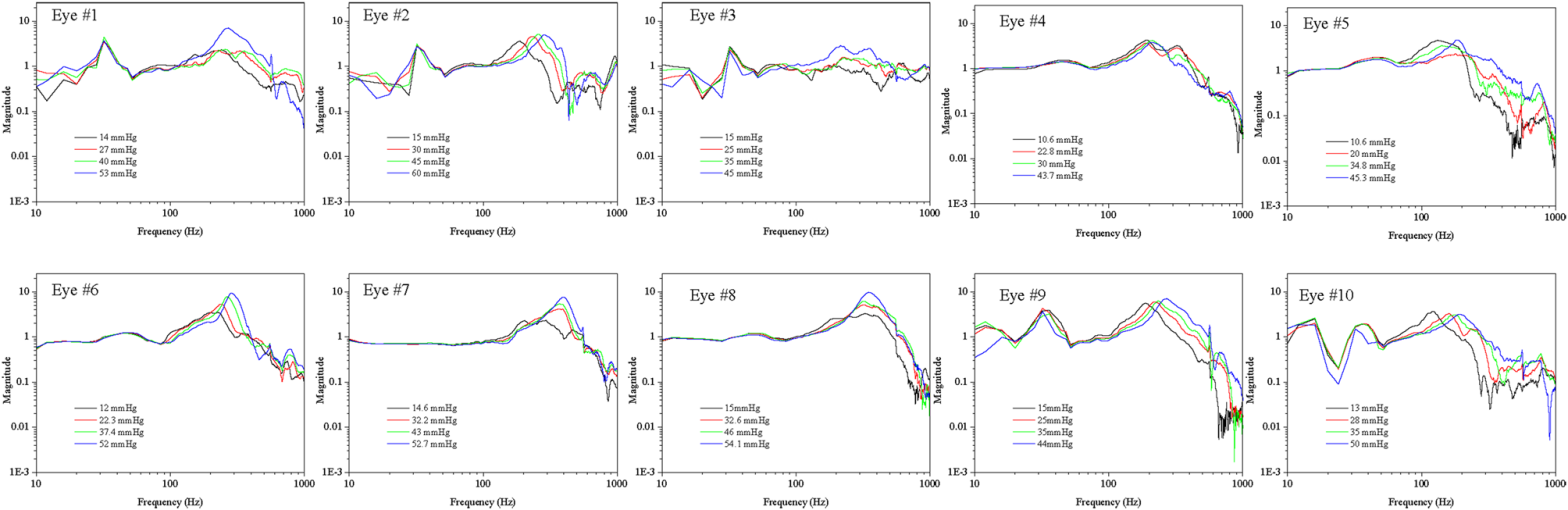
Transfer function according to increases in IOP Eye #1–#10.

Figure 1 shows the results of the measured transfer functions reflecting IOP increases in the porcine eyes 1–10. These measurements showed that the transfer function graphs differ among the porcine eyes. However, in all eyes, the resonance frequencies shifted to high frequency ranges with increasing IOP values but the degree of change in resonance frequency with IOP increase was different for each eyeball. Table 1 shows the result of resonance frequency of the eyes according to the IOP. For eye 1, when the IOP was increased from 14 to 53 mmHg, the resonance frequency shifted from 224 to 276 Hz; for eye 7, when the IOP increased from 14.6 mmHg to 52.7 mmHg, the resonance frequency shifted from 243 to 400 Hz. The magnitude of the increase in IOP in the two eyes was similar, but the degree of resonance frequency shifting was significantly different. This seems to be due to the fact that all of the experimental eyes differed in terms of weight and characteristics and because the boundary conditions and initial IOPs differed for each eye under the current experimental conditions.

**Table 1:** Resonance Frequency Changes with Changes in IOP.

Experiment case	IOP (mmHg)			
	Resonance frequency (Hz)			
Eye 1	14	27	40	53
	224	228	264	276
Eye 2	15	30	45	60
	187	230	264	283
Eye 3	16	30	40	52
	188	228	252	276
Eye 4	10.6	22.8	30	43.7
	194	200	206	212
Eye 5	10.6	20	34.8	45.3
	132	136	148	188
Eye 6	12	22.3	37.4	52
	200	244	268	288
Eye 7	14.6	32.2	43	52.7
	243	368	382	400
Eye 8	15	32.6	46	54.1
	322	322	322	353
Eye 9	15	25	35	45
	186	215	236	269
Eye 10	13	28	35	50
	120	160	176	192

*IOP* intraocular pressure.

### Numerical analysis of eyeball vibration behavior

To confirm the validity of these measured IOP-induced changes in resonance frequency, the finite element method (FEM) interpretation program ANSYS 17.2 (Ansys, Inc., Canonsburg, PA, USA) was used for a simulation with a simple numerical analysis model that describes the eyeball. The sclera and retina were described in this model as one circular rubber shell. To replicate the increases in IOP, the pressure inside this model eye was increased during the simulation.

**Figure 2:**
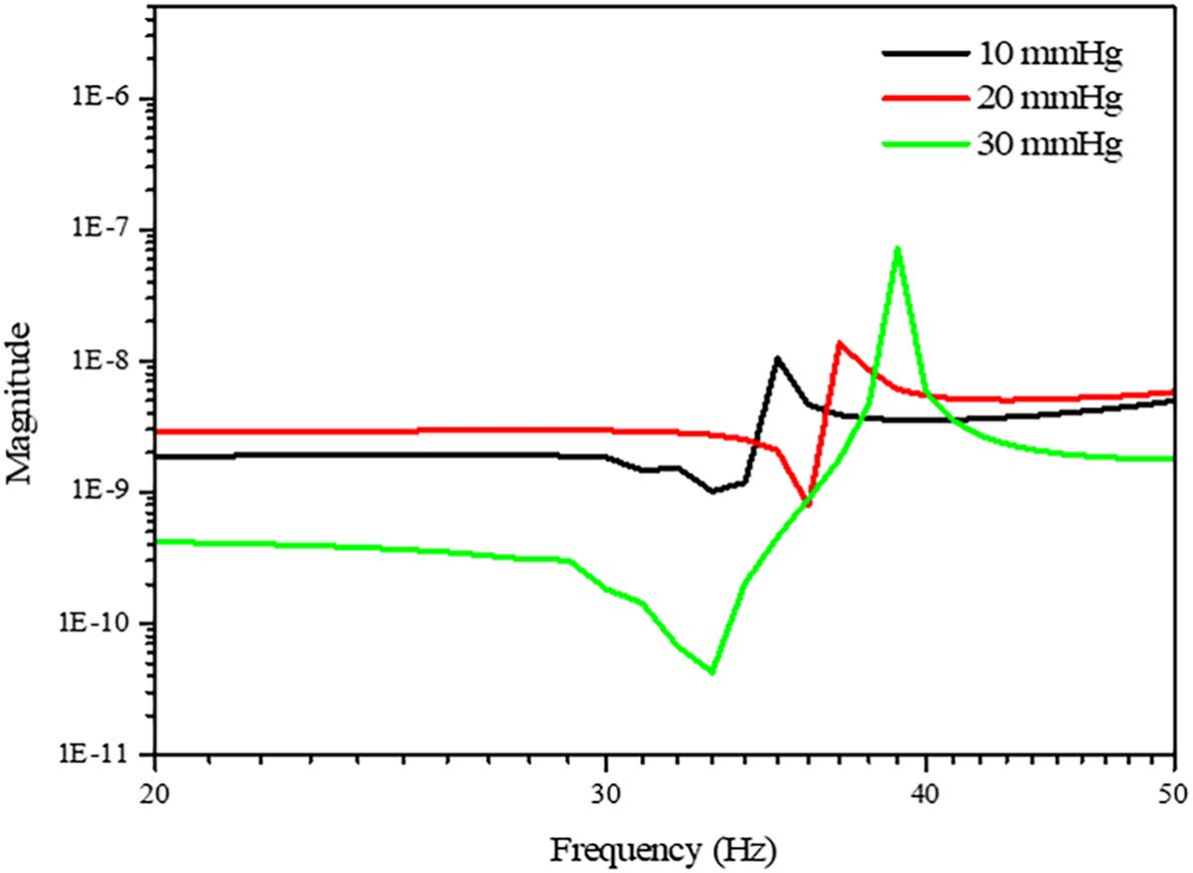
FEM analysis result of the eyeball vibration responses according to IOP increases.

Figure 2 shows the results of the transfer functions according to increases in the internal pressure. These results demonstrated that the resonance frequency of 35 Hz at 10 mmHg changed to 39 Hz when the IOP was elevated to 30 mmHg. Although the determined frequency values differ between simulated and experimental conditions, the result that IOP increases are accompanied by higher resonance frequencies is consistent.

### Effects of IOP on spectral characteristics

**Figure 3:**
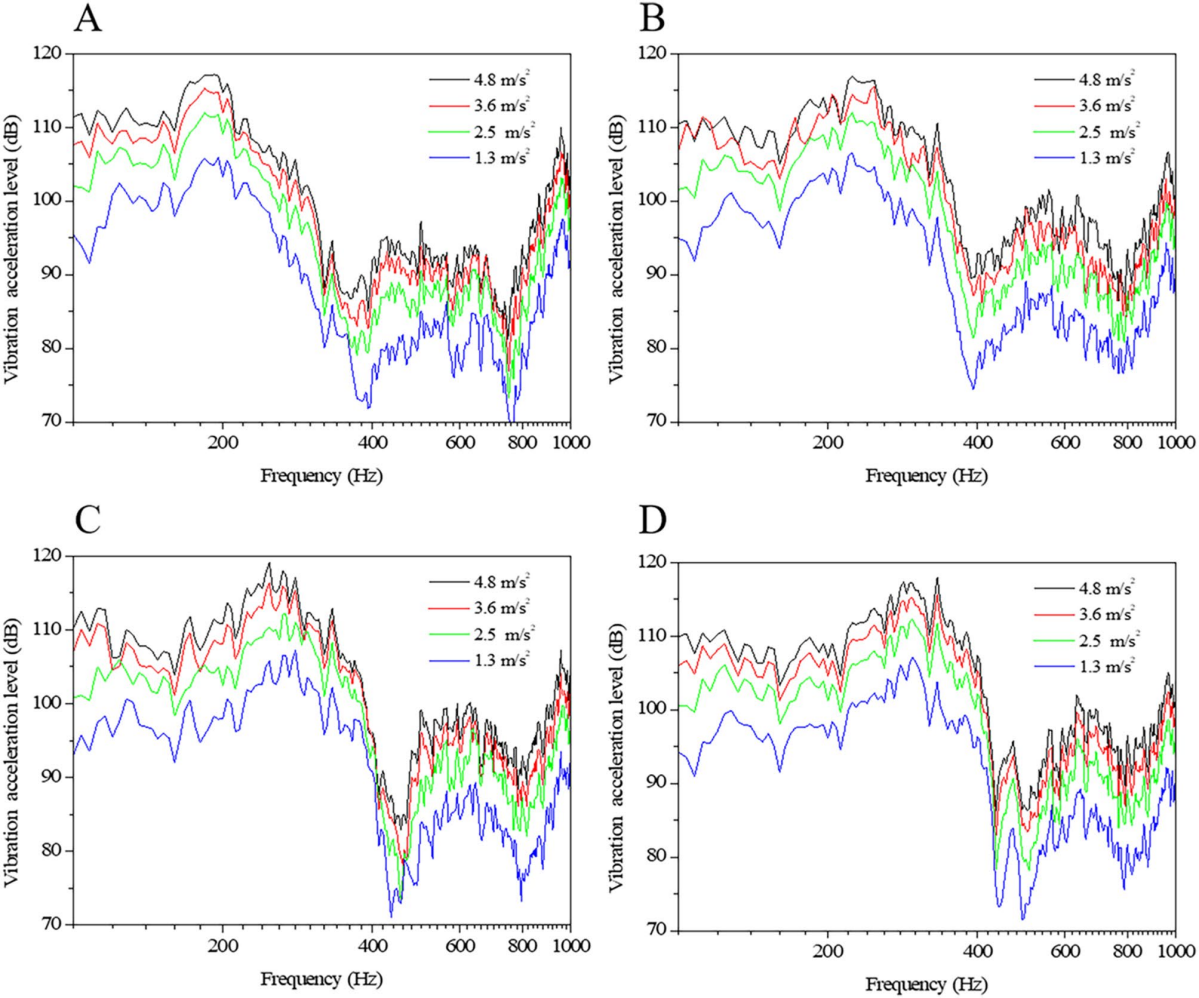
Auto-spectrum responses of eyeball 2 according to varying excitation and IOP levels (**A**) IOP 15 mmHg (**B**) IOP 30 mmHg (**C**) IOP 45 mmHg (**D**) IOP 60 mmHg.

Although the resonance frequency of an eyeball always increases with increased IOP values, the range of the resonance frequency changes differs since the initial resonance frequency is different for each eyeball. Therefore, it is difficult to measure the IOP using the resonance frequency. To determine a vibroacoustic property that shows an identical tendency in all eyes with increases in IOP, the results of the auto-spectrum analysis that can represent the vibration energy applied to an eyeball were compared. Figure 3 shows the results of the auto-spectrum analyses of eyeball 2 calculated for different IOP values and varying excitation outputs of the same vibration signal. The results demonstrated that the vibration response in frequency ranges above 300 Hz increased at higher IOP values and that the vibration response varies for large output signals. To show the magnitude of vibration responses in frequency ranges above 300 Hz in a single value, a 300-Hz high-pass filter was applied to the data, and root mean square (RMS,$x_{RMS}$) levels were calculated.

Table 2 summarizes the RMS levels according to excitation magnitude and IOP values for all eyes at frequency ranges above 300 Hz. In all eyeballs, the RMS levels tended to increase with elevated IOP values. Similarly, with higher IOPs, the differences in RMS levels according to the excitation magnitude increased. Thus, change in the magnitude of the vibration response (CMVR,$\Delta \upsilon$) quantified the changes in RMS levels with increasing IOP values according to Eq. (1).

1 $$\Delta \upsilon = x_{RMS} (a_{1} ) - x_{RMS} (a_{4} )$$

**Table 2:** RMS levels according to excitation magnitude and IOP.

Eye ball case	IOP (mmHg)	$$\begin{gathered} x_{RMS} (a_{n} ) \, \hfill \\ {\text{ (m/s}}^{2} ) \hfill \\ \end{gathered}$$				$$\begin{gathered} \, \Delta \upsilon \hfill \\ (m/s^{2} ) \hfill \\ \end{gathered}$$
		$$\begin{gathered} a_{1} = 4.8 \hfill \\ {\text{ (m/s}}^{2} ) \hfill \\ \end{gathered}$$	$$\begin{gathered} a_{2} = 3.6 \hfill \\ {\text{ (m/s}}^{2} ) \hfill \\ \end{gathered}$$	$$\begin{gathered} a_{3} = 2.5 \hfill \\ {\text{ (m/s}}^{2} ) \hfill \\ \end{gathered}$$	$$\begin{gathered} a_{4} = 1.3 \hfill \\ {\text{ (m/s}}^{2} ) \hfill \\ \end{gathered}$$	
1	14	1.8	1.6	1.2	0.6	1.2
	27	3.3	2.6	1.7	0.9	2.4
	40	3.8	2.9	2	1.1	2.7
	53	4.3	3.3	2.3	1.2	3.1
2	15	2.2	1.7	1.1	0.6	1.6
	30	2.7	2	1.4	0.7	2
	45	3.4	2.7	1.9	1	2.4
	60	4.8	3.6	2.5	1.2	3.6
3	16	2.2	1.9	1.4	0.8	1.4
	30	3	2.4	1.7	0.9	2.1
	40	3.2	2.7	1.9	1	2.2
	52	3.9	3.2	2.2	1.2	2.7
4	10.6	0.92	0.88	0.65	0.37	0.55
	22.8	2.06	1.49	1.05	0.58	1.48
	30	2.31	1.8	1.13	0.56	1.75
	43.7	4.63	3.6	2.51	1.29	3.34
5	10.6	1.46	1.36	1.27	0.73	0.73
	20	2.29	2.18	1.99	1.14	1.15
	34.8	4.22	3.34	2.38	1.36	2.86
	45.3	4.81	3.75	2.82	1.4	3.41
6	12	1.55	1.17	0.91	0.53	1.02
	22.3	2.71	2.5	1.45	0.81	1.9
	37.4	3.84	3.07	2.22	1.16	2.68
	52	4.77	4.02	2.54	1.31	3.46
7	14.6	1.63	1.38	0.85	0.52	1.11
	32.2	3.4	2.98	1.79	0.76	2.64
	43	4.05	3.17	2.2	0.88	3.17
	52.7	5.24	4.09	2.83	1.5	3.74
8	15	2.16	1.86	1.31	0.89	1.27
	32.6	3.25	2.84	1.94	1.09	2.16
	46	4.18	3.49	2.65	1.4	2.78
	54.1	5.08	4.11	3.13	1.63	3.45
9	15	1.76	1.75	1.29	0.5	1.26
	25	3.25	2.93	2.24	1.4	1.85
	35	4.49	3.98	2.96	2.13	2.36
	45	6.73	6.24	4.95	3.9	2.83
10	13	2.12	1.79	1.63	0.7	1.42
	28	3.4	3.01	1.94	1.09	2.31
	35	3.92	3.42	2.59	1.3	2.62
	50	4.5	3.68	2.75	1.47	3.03

*IOP* intraocular pressure, *RMS* root mean square, $x_{RMS} (a_{n}^{{}} )$ acceleration root mean square in eyeball, $a_{n}$ acceleration root mean square in excitation {n = 1,2,3,4}, $\Delta \upsilon$ = change in the magnitude of the vibration response.

**Figure 4:**
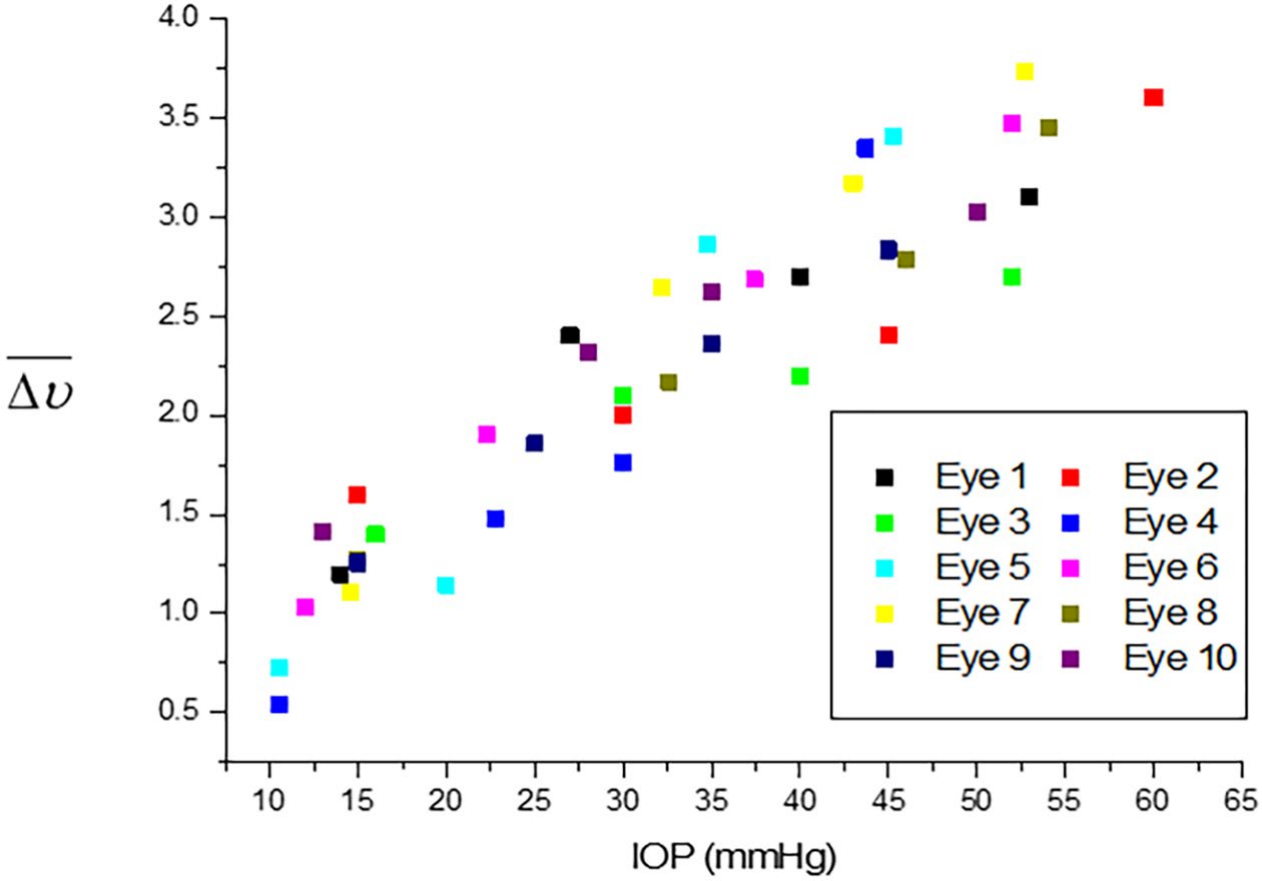
CMVR in relation to the IOP.

Figure 4 shows the CMVR changes according to IOP increases in all ten porcine eyes. The CMVR values tended to increase with higher IOP values.

**Figure 5:**
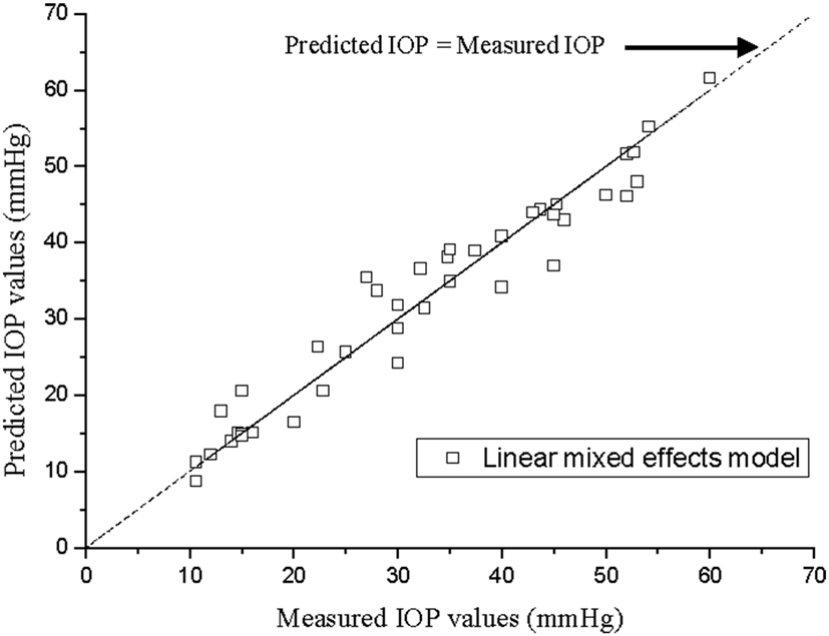
The measured IOP values against the predicted IOP values by LMM. Statistical analysis result was created by the authors using MATLAB R2019b (https://kr.mathworks.com/products/new_products/release2019b.html).

The LMM was used as a statistical analysis method to calculate the correlation between IOP and CMVR. Table 3 shows the estimated results and regression coefficients regarding CMVR in the LMM. Figure 5 shows the measured IOP values against the IOP values predicted by the LMM. In LMM analysis, CMVR was significantly correlated with IOP (p-value < 0.0001).

**Table 3:** Estimated results and regression coefficients regarding CMVR in LMM.

Fixed effects	Estimate	SE	p-value
Intercept	− 6.3356	2.8755	0.0337
CMVR	17.247	1.4444	< 0.001

*CMVR* change in the magnitude of the vibration response, *LMM* linear mixed effects model.

## Discussion

This study is a pilot study on vibroacoustic properties that can be used to infer IOP. In order to find vibroacoustic properties associated with IOP, experiments to measure vibration responses of the eye were conducted. These responses were analyzed to identify the vibroacoustic parameters associated with IOP.

The first vibroacoustic property associated with IOP is the resonance frequency. In all examined eyeballs, the resonance frequency increased with increasing IOP values. The resonance frequency, an inherent vibroacoustic property of an object, is determined by the boundary condition, material, and size. This is related to increases in the elastic modulus inside the eyeball resulting from increases in IOP.

2 $$\sigma = \frac{PR}{{2t}}$$

According to Laplace’s law shown in Eq. (2), the stress σ applied to the cornea can be calculated using the intraocular pressure P, cornea radius R, and cornea thickness t^19^. Thus, stress increases when the IOP increases. Stress and strain are in a nonlinear relationship in the cornea, and the elastic modulus, thus, increases with elevated stress values^26^. The linear relationship between stress and elastic modulus was verified by previous studies. When the elastic modulus of an object increases, the rigidity increases, thereby increasing the resonance frequency. Therefore, our experimental finding that the resonance frequency increased with elevated IOP levels is valid. However, the range of resonance frequency changes and the magnitude of change in resonance frequency with increases in IOP differed for each eyeball. This is because the resonance frequency of an eyeball is influenced by Poisson’s ratio, density, and radius, in addition to elastic modulus^22^. Therefore, measuring the IOP only based on the resonance frequency of an eyeball has its limitations.

CMVR is another vibroacoustic parameter associated with IOP. CMVR has a negative linear relationship with vibration reduction property. When an object's vibration reduction property decreases, the CMVR, which results from differences in the magnitude of the applied vibration, increases. By contrast, when the vibration reduction property is large, the CMVR resulting from differences in the magnitude of the applied vibration decreases. Our experimental data confirmed that CMVR, which can represent vibration reduction properties, is highly correlated with IOP. Moreover, we confirmed that IOP can be measured using an LMM based on CMVR.

In contrast to previous studies that used noninvasive ultrasonography to measure vibroacoustic properties in relation to changes in IOP^19–21^, we attached an accelerometer directly on the eyeball to measure the vibroacoustic properties. Since the responses were measured through the attached accelerometer, the experiments did not have many limitations. Since the responses were directly measured from the eyeballs, the results are highly reliable. Furthermore, the frequency range analyzed in the present study is lower than the ultrasound range, so the results are more applicable.

There are many commercially available tonometers. The GAT and Tono-Pen are based on applanation, where the cornea is anesthetized and pressed to infer the IOP from the resulting changes. This potentially invasive method is influenced by corneal thickness, curvature, and other biomechanical properties. A rebound tonometer uses the principle of induction and impact. The probe hits the cornea and returns to the original position, and the deceleration time for the probe at the time of impact is converted into the IOP value. This rebound tonometer made up for a lot of the shortcomings of GAT as it does not require anesthesia and is easy to use. However, since the measurement must be taken with the eye open, the patient may experience fear, and it can feel invasive due to brief but intimate contact with the cornea. The need to change the tip, which is a consumable, can be inconvenient and costs can steadily accumulate. The ocular response analyzer and the Corvis tonometer, which both take biomechanical factors into consideration, have been recently introduced. Here, air pressure is applied resulting in cornea deformation, and time measurements or images are acquired. As such, most tonometers apply physical pressure through direct contact with the cornea or cause indentations of the cornea through the air, and these changes are analyzed subsequently. In the present study, we used vibration as the stimulus, and CMVR, a novel parameter, was used to infer the IOP. Since this method uses noninvasive stimuli, namely sound wave and vibration, it is safe for patients. Recently, contact lens tonometers were introduced to assess the nychthemeral IOP. However, contact lens tonometers are still expensive and are inconvenient for those unfamiliar with wearing lenses, such as children and the elderly. In addition, it is difficult to see steady changes because it can only be used once within 24 h. If the vibration application and CMVR measurement can be simplified through technological advances, IOP measurements may also become easier, e.g., by applying vibration to the closed eyelid and calculating the IOP from the measured responses. Moreover, as the CMVR is derived from the accelerometer response made by piezoelectric material, it would be possible to continuously measure the IOP with a lower cost compared to the existing method. This will facilitate IOP measurements in patients who are not compliant, and self-monitoring of IOP at home may also become possible. It can also act as a substitute for measuring nychthemeral IOP when applied in the form of goggles with technology development in the future.

A limitation of the present study is the small sample size. The present study aimed to confirm the possibility of using vibroacoustic properties to measure the IOP. It is significant for a pilot study to find appropriate parameters. Therefore, the parameters identified in the present study will need to be explored further in future studies. Second, although the IOP was measured multiple times with a Tono-Pen and the mean value was determined, the Tono-Pen itself may have been inaccurate. We used human instead of veterinary Tono-Pens, which may have led to inaccurate measurements. However, multiple measurements were made to obtain the mean, and the height of the saline bottle was adjusted to overcome inaccuracies through relative differences. Third, in this experiment, the central corneal thickness value was not measured or modified. This work was a pilot stage study, in which an accelerometer was directly attached to the eyeball to measure the IOP. In the future, the technology will be developed to continuously measure the IOP through the vibration response measured on the eyelid with the eye closed.

In conclusion, in this pilot study using porcine eyes, we were able to confirm that resonance frequency and CMVR are vibroacoustic properties related to IOP. Of these, CMVR had a linear correlation with IOP. Therefore, CMVR is expected to serve as a new parameter for IOP measurements.

## Methods

### Sample preparation for vibration tests

Ten fresh porcine eyeballs were collected from slaughterhouses of the Majang Meat Market (Seoul, South Korea). Until the start of the experiments, the eyeballs were stored at 4 °C in a small, sealed Styrofoam box with all peribulbar fat intact to maintain hydration. All fat and peribulbar tissues except the extraocular muscles were subsequently removed. The optic nerve was trimmed flush to the sclera. The eyeballs were moistened with a balanced salt solution (BSS) to maintain hydration. All methods were carried out in accordance with relevant guidelines and regulations. As this study was conducted using only animal carcasses, and without using human participants, human tissue, or live animals, the need for obtaining approval from the institutional animal care and use committee was waived.

### Experimental procedures to measure eyeball vibrations

**Figure 6:**
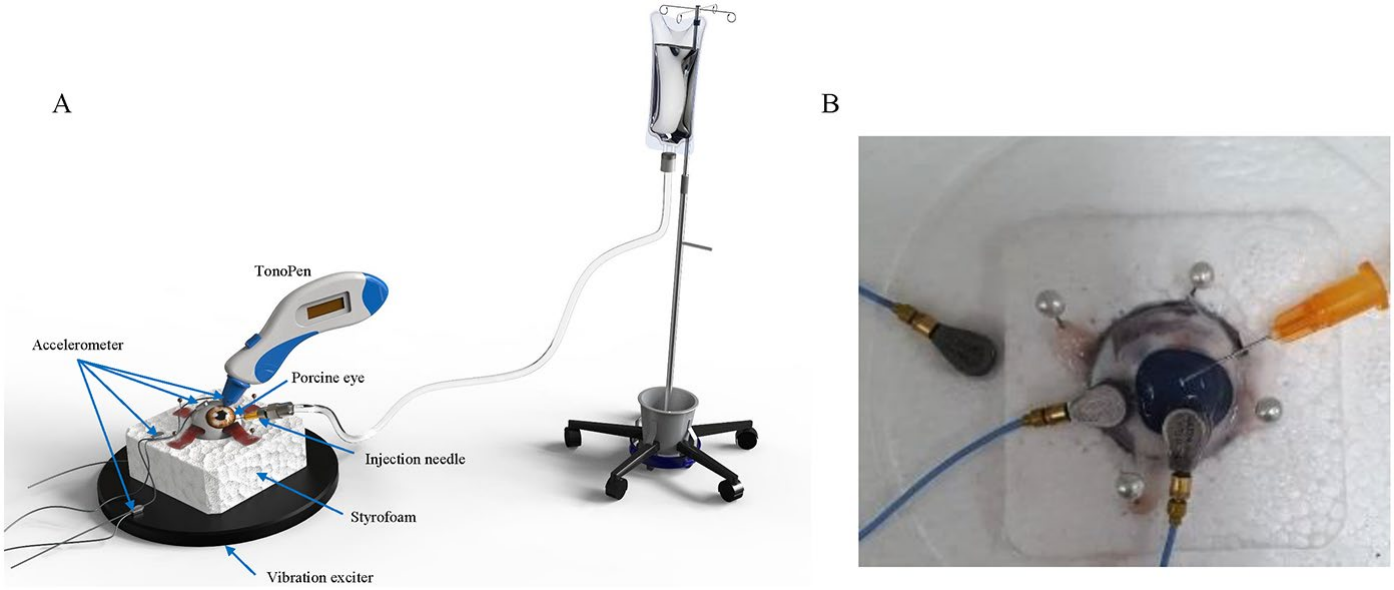
Experimental setup to detect vibration response changes induced by increases in IOP (**A**) Schematics of the experiment. (**B**) Photograph of the experimental porcine eye.

To measure the vibroacoustic properties of eyeballs in response to changes in IOP, an experimental setup as shown in Fig. 6A was established. In the experiment, ex vivo porcine eye as shown in Fig. 6B was used. A Styrofoam block holding the eyeball is attached to a vibration exciter (44MM vibration speaker; AIYIMA). To stabilize the eyeball in the same position while it expands due to increases in IOP, it was placed in a hole in the center of the Styrofoam block. Four pins were inserted into the extraocular muscles and the Styrofoam box to secure the eyeball. The vibration exciter applied vertical excitation to the porcine eye through the Styrofoam block. Accelerometers (352A21 miniature accelerometers; PCB) were placed on the cornea of the porcine eye, the Styrofoam block, and the vibration exciter to measure vibration responses. All vibration experiments were performed on a vibration isolation table to minimize the influence of external noise and vibration. The sampling frequency was 16,384 Hz for 1 s, and the magnitude of excitation in Gaussian random noise was varied. To control the IOP, a 30-gauge needle of the infusion line connected with the BSS was inserted into the anterior chamber of the porcine eyes through the limbus. The IOPs were changed according to the height of the connected infusion bottle. To change the IOP, the height of the infusion bottle was adjusted in 4 steps in each eye (20, 40, 60, and 80 cm from the needle). A hand-held tonometer (Tono-Pen; Reichert, Inc.) was used for IOP measurements under identical conditions, and the mean value of five measurements was used for further analyses.

### Vibration analysis used to extract spectral characteristics

Since the magnitude of vibration changes over time, signal processing was used to analyze the time domain response. To obtain the magnitude of the vibration response applied during the measurement, the acceleration RMS was calculated in the time domain according to Eq. (3). Here, x(t) is the accelerometer measurement in the time domain, and T is the measurement time.

3 $$x_{RMS} = \left( {\frac{1}{T}\int_{0}^{T} {x^{2} (t)dt} } \right)^{1/2}$$

To analyze the frequency characteristics of a measured response, the response in the time domain was converted to the frequency domain. Thus, an auto-spectrum ($S_{xx} (\omega )$) according to Eq. (4) was used to obtain the vibration magnitude in each frequency spectrum in the measured vibration response. Here, τ is the time difference, and $\omega$ is angular frequency.

4 $$S_{xx} \left( \omega \right) = \int {x(t)x(t + \tau )e^{ - i\omega \tau } } d\tau$$

To explore the frequency characteristics of the vibration response transmitted from the excitation signal, the transfer function *H*(*ω*) as shown in Eq. (5) was calculated for further analyses. Here, *X*(*ω*) is the frequency response of the excitation signal, and *Y*(*ω*) is the frequency response of the measured signal.

5 $$H(\omega ) = \frac{X(\omega )}{{Y(\omega )}}$$

### Eyeball analysis using FEM model

**Figure 7:**
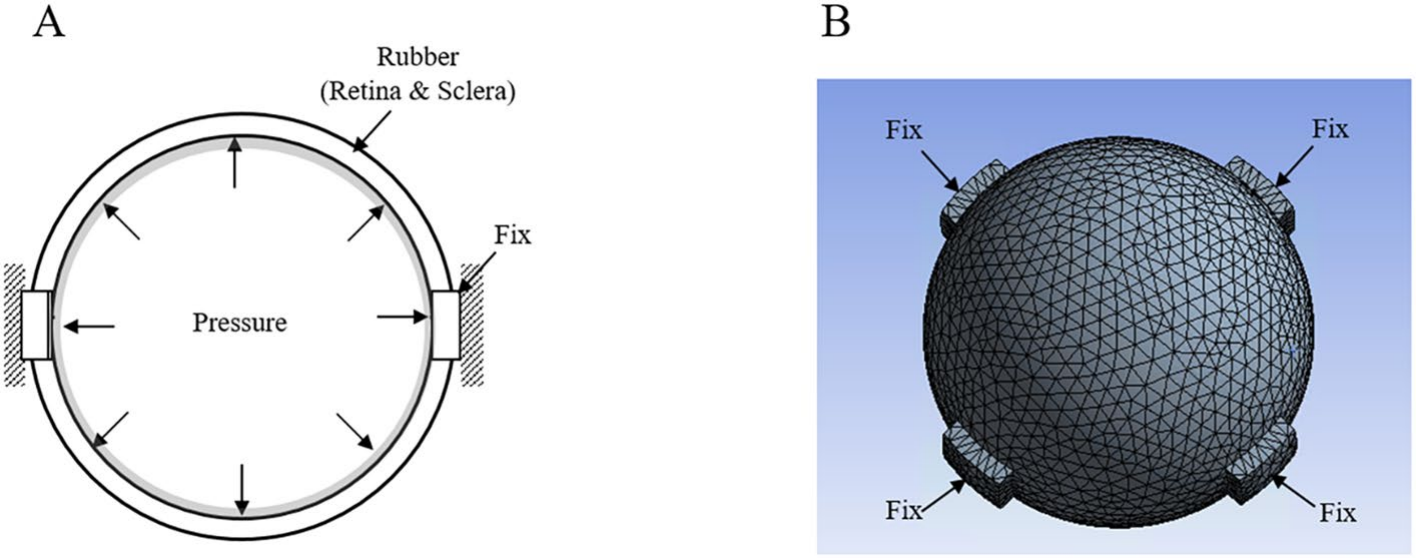
Eyeball FEM analysis (**A**) Eyeball analysis model (**B**) Mesh of the eyeball analysis model. The mesh model was developed using ANSYS 17.2 (http://www.ansys.com).

FEM analysis was performed to verify the porcine eye experiment results. A 3-dimensional eyeball model was designed and meshed using ANSYS Workbench. Figure 7A shows the eyeball analysis model. The eye model was modeled as a 0.5 mm thick spherical shell with a 30 mm diameter and an elastic modulus of 0.25 MPa. Figure 7B shows the mesh of the eyeball analysis model. The mesh model was developed using ANSYS. The eyeball mesh file was then transferred and simulated using ANSYS. Fixed condition was applied as the boundary condition. The IOP was described as applying positive pressure inside the spherical shell. Harmonic response analyses were performed to obtain the eyeball transfer function.

### Statistical analysis between CMVR and IOP

The experimental results of this study measured CMVR when the IOP in the same eye was increased. To find the correlation between IOP and CMVR, LMM was used for statistical analysis. LMM is a statistical model containing both fixed effects and random effects^27,28^. The software used for statistical analysis is MATLAB R2019b (The MathWorks, Inc., Natick, Massachusetts, USA).
